# Haemothorax in Military Practice

**Published:** 1916-05

**Authors:** 


**Haemothorax in Military Practice.** Sir J. R. Bradford and Capt. T. R. Elliott, Brit. Jour. of Surg., p. 247, 1915, vol. 3, report 168 cases, Nov. 1914-March 1915, and 160 cases March-

July 1915, the latter series including only severe cases. 114 of the first group and 86 of the second were sterile. 28 infected cases survived after resection of a rib in the first series, 53 in the second series. The total mortality of chest wounds reaching the base hospitals is 10%. Simple haemorrhage never causes death after the third day, the danger after this time being mainly from sepsis. Primary infection occurs in about 25% of effusions and is fatal in about a third of the infected cases. It should be suspected in cases not progressing favorably after the fourth day, but can be diagnosed positively only by bacteriologic examination of a specimen aspirated. Sterile haemothorax should be aspirated if the effusion extends more than half way up the scapula. A complete pneumothorax or large pneumohaethorax due to leak of air from the lung is not often infected. Immediate resection should be performed if infection occurs, aspirating first if air is also present.

